# Involuntary reflexive pelvic floor muscle training in addition to standard training versus standard training alone for women with stress urinary incontinence: study protocol for a randomized controlled trial

**DOI:** 10.1186/s13063-015-1051-0

**Published:** 2015-11-17

**Authors:** Helena Luginbuehl, Corinne Lehmann, Jean-Pierre Baeyens, Annette Kuhn, Lorenz Radlinger

**Affiliations:** Bern University of Applied Sciences, Health, Discipline of Physiotherapy, Bern, Switzerland; Vrije Universiteit Brussel, Faculty of Physical Education and Physiotherapy, Brussels, Belgium; Department of Physiotherapy, Bern University Hospital and University of Bern, Bern, Switzerland; Women’s Hospital, Urogynaecology, Bern University Hospital and University of Bern, Bern, Switzerland

**Keywords:** Electromyography, Exercise, Muscle contraction, Pelvic floor, Reflex

## Abstract

**Background:**

Pelvic floor muscle training is effective and recommended as first-line therapy for female patients with stress urinary incontinence. However, standard pelvic floor physiotherapy concentrates on voluntary contractions even though the situations provoking stress urinary incontinence (for example, sneezing, coughing, running) require involuntary fast reflexive pelvic floor muscle contractions. Training procedures for involuntary reflexive muscle contractions are widely implemented in rehabilitation and sports but not yet in pelvic floor rehabilitation. Therefore, the research group developed a training protocol including standard physiotherapy and in addition focused on involuntary reflexive pelvic floor muscle contractions.

**Methods/design:**

The aim of the planned study is to compare this newly developed physiotherapy program (experimental group) and the standard physiotherapy program (control group) regarding their effect on stress urinary incontinence. The working hypothesis is that the experimental group focusing on involuntary reflexive muscle contractions will have a higher improvement of continence measured by the International Consultation on Incontinence Modular Questionnaire Urinary Incontinence (short form), and — regarding secondary and tertiary outcomes — higher pelvic floor muscle activity during stress urinary incontinence provoking activities, better pad-test results, higher quality of life scores (International Consultation on Incontinence Modular Questionnaire) and higher intravaginal muscle strength (digitally tested) from before to after the intervention phase. This study is designed as a prospective, triple-blinded (participant, investigator, outcome assessor), randomized controlled trial with two physiotherapy intervention groups with a 6-month follow-up including 48 stress urinary incontinent women per group. For both groups the intervention will last 16 weeks and will include 9 personal physiotherapy consultations and 78 short home training sessions (weeks 1–5 3x/week, 3x/day; weeks 6–16 3x/week, 1x/day). Thereafter both groups will continue with home training sessions (3x/week, 1x/day) until the 6-month follow-up.

To compare the primary outcome, International Consultation on Incontinence Modular Questionnaire (short form) between and within the two groups at ten time points (before intervention, physiotherapy sessions 2–9, after intervention) ANOVA models for longitudinal data will be applied.

**Discussion:**

This study closes a gap, as involuntary reflexive pelvic floor muscle training has not yet been included in stress urinary incontinence physiotherapy, and if shown successful could be implemented in clinical practice immediately.

**Trial registration:**

NCT02318251; 4 December 2014

First patient randomized: 11 March 2015

**Electronic supplementary material:**

The online version of this article (doi:10.1186/s13063-015-1051-0) contains supplementary material, which is available to authorized users.

## Background

Stress urinary incontinence (SUI), the most common urinary incontinence subtype in women with a prevalence of 24.8 % [1], is defined as involuntary loss of urine during effort or physical exertion (for example, during sporting activities) or upon sneezing or coughing [2]. Activities typically provoking SUI raise the intra-abdominal pressure and impact loading on the pelvic floor muscles (PFM) and require strong, rapid, and reflexive PFM contractions to maintain continence [3–6]. Fast and strong PFM contractions result in the generation of an adequate squeeze pressure in the proximal urethra, which maintains a pressure higher than that in the bladder, thus preventing leakage [7]. PFM function regarding power (rate of force development) is impaired in incontinent women compared to continent women [4, 6].

PFM training — defined as a program of repeated voluntary PFM contractions taught and supervised by a health care professional — is the most commonly used physiotherapy treatment for women with SUI, is effective with all types of female incontinence, and, therefore, is recommended as a first-line therapy [8, 9]. As endorsed by the International Consultation on Incontinence, PFM training should generally be the first step of treatment before surgery [10]. However, standard SUI physiotherapy concentrates on voluntary contractions even though the situations provoking SUI such as sneezing, coughing, jumping, and running [2] require involuntary fast reflexive PFM contractions [4]. Although training procedures following the concepts of training science and sports medicine are generally well known and widely implemented in rehabilitation and sports [11, 12], an optimal and well-standardized training protocol for involuntary, fast, and reflexive PFM contractions still remains unknown.

Consequently, the research group of the present study developed a standardized therapy program, which includes the standard physiotherapy and additionally focuses on involuntary fast reflexive PFM contractions. The additional exercises are well known and applied in physiotherapy, however not yet regarding SUI.

Therefore, the aim of the present study is to compare two different physiotherapy programs: standard training plus involuntary reflexive PFM training versus standard training alone, regarding their effect on SUI and the impact of incontinence symptoms on quality of life. Both programs include standard physiotherapy. Both follow the concepts of training science, that is, periodization/exercise sequence and training of specific muscle strength components [11, 12]. One program focuses on voluntary fast contractions (standard physiotherapy; control group); the other one additionally focuses on involuntary fast reflexive PFM contractions (experimental group). The secondary objective, based on secondary and tertiary outcomes, is a deeper insight into the functioning of the PFM (PFM activity characteristics and muscle action forms) by evaluating their activity measured by electromyography (EMG) in the quality of life of patients with SUI and in patients’ therapy adherence.

## Methods/design

### Study design

The present study is a single-centered, prospective, triple-blinded (participant, investigator, outcome assessor), parallel group, non-inferiority randomized controlled trial with two physiotherapy intervention groups with a 6-month follow-up.

### Blinding

Participants will be blinded against the type of received physiotherapy (control group, experimental group). The participant information document will not provide any information regarding the differences in the experimental and control therapy protocols in a way that women could find out about their group allocation. All investigators involved in data acquisition, data reduction, data analyses, and statistics will also be blinded against group allocation. The physiotherapists in charge of the therapy cannot be blinded against group allocation and therefore will not be involved in data acquisition, data reduction, data analyses, or statistics.

### Participants

With ethics committee approval (Ethics Committee of the Canton of Bern reference number 249/14 on 12 November 2014; Chairperson Prof. Dr. Ch. Seiler), which is in accordance with the Declaration of Helsinki and the Swiss Human Research Act, and written informed consent, patients will be included on the condition that they are female adults aged 18–70 years, suffer from SUI (based on the patient’s history) or mixed urinary incontinence (with dominant SUI), are one year post-partal, parous, nulliparous, pre- or post-menopausal, have a BMI of 18–30 kg/m^2^, are medically and physically fit for the measurement and therapeutic exercises (running, jumps), and, in case of systemic or local estrogen treatment, stable for the past 3 months prior to inclusion.

Exclusion criteria are: urge incontinence or predominant urgency in incontinence; prolapse > grade 1 POP-Q [13] (uterus, cystocele, rectocele during Valsalva maneuver); pregnancy (urine test to accomplish); current urinary tract or vaginal infection, menstruation on the day of examination; lactation period not yet finished; contraindications for measurements or interventions, for example, acute inflammatory or infectious disease, tumor, fracture; de novo systemic or local estrogen treatment (<3 months); de novo drug treatment with anticholinergics or other bladder active substances (tricyclic antidepressants, selective serotonin reuptake inhibitors). Interventions and measurements would be individually modified in case of temporary loss of physical fitness for certain exercises.

### Outcomes

#### Primary outcome measure

The primary outcome measure will be the International Consultation on Incontinence Modular Questionnaire Urinary Incontinence short form (ICIQ-UIsf), which provides a brief and robust assessment of the impact of symptoms of urinary incontinence on quality of life and outcome of treatment and is scored on a scale from 0 (not) – 21 (severely affected). Previous research has examined the ICIQ-UIsf questionnaire and found it to have good reliability and constructive validity [14] and to correlate well with urodynamic findings [15]. This questionnaire is validated in the German language [16]. For measurement time points see Table 1.

**Table 1:** Outcome measures and personal physiotherapy consultation time points

	Baseline before intervention phase	16-week intervention phase (1) after randomization into control group and experimental group									After intervention phase	6-month follow-up
Visits	1	2	3	4	5	6	7	8	9	10	11	12
Weeks	−1	1	2	4	6	8	10	12	14	16	16 + 1	16 + 26
Personal Physiotherapy Consultation (PT)		PT1	PT2	PT3	PT4	PT5	PT6	PT7	PT8	PT9		
Primary outcome												
ICIO-UIsf	x		x	x	x	x	x	x	x	x	x	
Secondary outcomes												
EMG (rest, MVC)	x										x	x
EMG (VFC, SJ, CMJ, DJ)	x										x	x
EMG (running 7, 9, 11 km/h)	x										x	x
ICIQ-UIsf												x
ICIQ-LUTSqol	x										x	x
20-min pad test	x										x	x
Tertiary outcomes												
Manual muscle testing	x										x	x
Therapy adherence			x	x	x	x	x	x	x	x	x	x

*ICIQ-UIsf* International Consultation on Incontinence Modular Questionnaire Urinary Incontinence (short form), *EMG* electromyography, *MVC* maximal voluntary contraction, *VFC* voluntary fast contraction, *SJ* squat jump, *CMJ* counter movement jump, *DJ* drop jump, *ICIQ-LUTSqol* International Consultation on Incontinence Modular Questionnaire Urinary Incontinence Symptoms Quality of Life

#### Secondary and tertiary outcome measures

The secondary outcome measure will be the EMG activity of the PFM during rest, maximal voluntary contractions (MVCs), voluntary fast contractions (VFCs), and during involuntary contractions during squat jumps (SJs), counter movement jumps (CMJs), drop jumps (DJs), and treadmill running at 7, 9, and 11 km/h [17]. As there is a relation between high impact activities and SUI prevalence [18], those different and comprehensive test exercises will be chosen to represent typical situations of involuntary urine loss and different muscle action forms (isometric-concentric voluntary muscle actions: MVC, VFC; involuntary eccentric-concentric muscle actions: SJ, CMJ, DJ, running).

Additionally, the 20-min pad test [19, 20] and the International Consultation on Incontinence Modular Questionnaire Urinary Incontinence Symptoms Quality of Life (ICIQ-LUTSqol) [21] will be implemented as secondary outcomes. The pad test will provide information regarding the efficacy of the therapy protocols to the UI symptoms (such as changes in the leakage volume) and the ICIQ-LUTSqol evaluates the impact on the participant’s quality of life with reference to social effects. It consists of 20 questions, which lead to a summary score between 19 and 76 points (greater values indicate higher impact on quality of life) and is available in German [22].

As tertiary outcomes PFM strength will be digitally assessed according to the Oxford Grading Scale [23], and adherence to the home exercise program will be assessed, that is, how many of the total therapy sessions will be completed individually. The home exercise adherence data will be collected with a simple training diary questionnaire.

For measurement time points, see Table 1.

### EMG procedure, instrumentation, and data reduction

#### Instrumentation

A vaginal surface EMG probe (3-pol-STIMPON® electrode (Innocept Biobedded Medizintechnik GmbH, Gladbeck, Germany) in a differential configuration will be used to measure PFM activity. The STIMPON electrode is a certified device (patent number EP 0 963 217 B1, CE 0482) which is widely applied in pelvic floor rehabilitation. The probe is made of polypropylene and with this soft surface therefore optimally designed to adapt its form to individual vaginal cavities and not to slip out of position during impact loads. The single reference adhesive surface electrode (Ambu Blue Sensor N, Ballerup, Denmark) will be fixed on the right iliac crest.

Running will be conducted on a Kettler Marathon TX1 treadmill device (Ense-Parsit, Germany) at speeds of 7, 9, and 11 km/h and 1° inclination. For the DJ a 20-cm step will be used.

To detect events necessary for the parameterization of the time-dependent signals such as heel strike (= initial contact = time point zero (T0)) during running, two load cells (Type KMB52 K 10KN, Megatron, Putzbrunn, Germany) are attached under the treadmill. T0 of SJ, CMJ, and DJ will be identified by using a force plate (Type 9286BA, Kistler Winterthur, Switzerland). Electrodes will be connected to the transmitter by short wire and the signals will be sent wirelessly to the receiver (TeleMyo 2400 G2, Noraxon European Service Center, Cologne, Germany).

#### EMG measurement procedures

The subjects will be instructed in the vaginal insertion of the surface EMG probe using ultrasound lubrication and will perform the insertion themselves after emptying their bladder. They will wear a loose running suit and running shoes of the same type but individual size. PFM EMG will be measured for 30 seconds (s) without any voluntary contraction and twice for 5 s during MVC (contraction maximal as possible), which will be instructed and controlled by intravaginal manual muscle testing before the EMG measurements, and ten times for VFC in a standing position, with a 15-s break between the single measurements. After that they will perform five SJs, five CMJs, and five DJs, again with 15 s between the single measurements, each jump being demonstrated and practiced before EMG recordings. Thereafter, the subjects will perform a warm-up of treadmill walking (5 km/h) for 30 s, then running at 7, 9, and 11 km/h consecutively until they reach a steady state. As soon as they reach the steady state at the respective speed, the data acquisition will be started: all data will be measured continuously for 10 s and the first ten step cycles of the right leg will be analyzed. Between the measurements of the different speeds the treadmill will be stopped, followed by a 1-minute break until the same procedure is restarted with the next speed [17].

#### Data reduction

All signals will be sampled at a rate of 3 kHz (sampling interval (dt) equals 0.333 ms) using a measurement amplifier and 1-bit analog-to-digital converter (ME-2600i, SisNova Engineering, Zug, Switzerland) and the software package “Analoge und digitale Signalverarbeitung” (ADS) version 1.12 (uk-labs, Kempen, Germany).

The EMG signals will firstly be first order high-pass filtered with a cut-off frequency of 10 Hz by EMG preamplifier leads to reject or eliminate artifacts and later digitally low-pass filtered by Noraxon Receiver Hardware with a cut-off frequency of 500 Hz. Secondly, to identify amplitude peaks during MVC, EMG will be calculated as RMS (200 ms moving window). 100 % of EMG equals the average of the two peak amplitude values during the two 5-s sessions of MVC. Thirdly, EMG variables of pre-activity and reflexivity will be calculated as RMS values within 30-ms intervals [24–26], averaged over 10 steps, and normalized to peak MVC (% EMG). The variables of pre-activity and reflexivity (30-ms intervals) will follow former study protocol intervals [17, 24, 25]. All variables will be analyzed using the software package MATLAB (MathWorks, Natick, MA, USA). Outcomes will be averaged over the repetitions of each exercise.

### Statistical methods

#### Hypothesis

Alternative hypothesis for primary outcome: It is hypothezised that the experimental group focusing on involuntary pelvic floor muscle contractions will have a statistically higher improvement of continence measured by ICIQ-UIsf questionnaire from before to after the intervention phase.

#### Sample size calculation

Sample size calculation was performed with G*Power software [27], using the statistical model of an ANOVA approach (repeated measures, within-between interactions). To this day there is no comparable training study (RCT) to estimate sample size based on available data from ICIQ-UIsf. Consequently, sample size was estimated theoretically and an effect size of = 0.1, indicating a small effect [28] will be accepted. The sample size was calculated for the primary outcome ICIQ-UIsf with the following assumptions: α = 0.05, power (1–β error probability) = 0.8, number of groups = 2, number of measurements = 10; correlations among repeated measures were estimated conservatively low with 0.5. Based on these assumptions, a total sample size of N = 80 was estimated. In anticipation of dropouts (10 %: n = 8) or a violation of normality assumption (+10 %: +n = 8), a final sample size of N = 96 (48 participants per group) results.

#### Statistical analyses

**Fig. 1:**
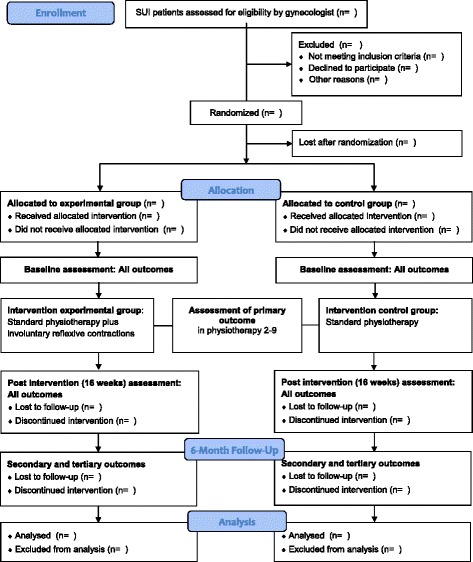
CONSORT study flow diagram

Analysis of the patients will follow the CONSORT flow diagram (see Fig. 1) through the phases of the study (enrollment (assessed, excluded, randomized), allocation (control group, experimental group with received intervention or not received intervention), follow-up (lost to follow-up, discontinued intervention), and analysis (excluded, included)) [29].

The outcomes of the control and experimental group will be analyzed by the repeated measures within-between interactions ANOVA approach and an intention-to-treat analysis (last observation carried forward method). No subgroup analyses are planned.

Primary analysis: For the descriptive analyses means, standard deviations, 95 % confidence intervals, and median, quartiles, minima, maxima will be used.

To compare the primary outcome ICIQ-UIsf between and within the two groups (control group, experimental group) at ten time points (before intervention, physiotherapy sessions 2–9, after intervention), ANOVA models for longitudinal data according to Brunner et al. [30] will be used. All statistical analyses will be realized after the final measurement of the last patient at the time point after intervention. The statistics will be calculated by SPSS and R software packages in the current versions. The repeated measure design with ten time points allows us to monitor how patients change over time, in both short-term (before/during intervention) and long-term situations (before/after intervention).

Secondary and tertiary analyses: All secondary and tertiary outcomes will be analyzed following the same approach and with the same statistical procedures as for primary outcomes. Generally, rational data will be checked for normality using Q-Q-plots. If the normality assumption is not violated, parametric ANOVA models for longitudinal data will be used [31]. As for intra-session retest procedures (EMG amplitude and frequency), data will also be described and checked for their reliability (ICC, SEM, MD, ANOVA).

### Detailed study plan

#### Patient recruitment/consent procedure

Patients will be recruited from the urogynecology consultation of the Women’s Hospital, Bern University Hospital and University of Bern, Switzerland. All participants for the study will be provided a participant information sheet and a consent form describing the study and providing sufficient information for them to make an informed decision about their participation in the study.

Each participant will be specifically informed that the participation in the study is voluntary and that she may withdraw from the study at any time and that withdrawal of consent will not affect her subsequent medical assistance and treatment.

#### Allocation of patients

Patients with written informed consent are randomly allocated to one of the two therapy groups (control group, experimental group). The allocation sequence will be generated by the independent urogynecology secretariat using online randomization software (http://randomization.com); allocation ratio = 1:1 (control group:experimental group). The allocation will be concealed in sealed, opaque, sequentially numbered envelopes, which will be stored at the secretariat and can be opened one at a time for each eligible patient. Physiotherapists treating patients will be informed about enrollment and group allocation of their patient by the independent secretariat.

#### Outcome measurements

Baseline (before intervention phase), after intervention phase, and 6-month follow-up measurements (of primary, secondary, and tertiary outcomes) will take place at the Bern University of Applied Sciences movement laboratory by two experienced pelvic floor physical therapists, who are blinded regarding group allocation of participants. Measurements during the 16-week intervention phase (ICIQ-UIsf; primary outcome) will be taken by the treating physiotherapists. However, to guarantee blinding of the outcome, the participant will fill in the questionnaire in the absence of the treating physiotherapist and seal it in an envelope, which will be given to the outcome assessor.

#### Intervention

Both therapy programs (control group, experimental group) are based on the latest position stand paper of the American College of Sports Medicine [11, 32], PFM motor learning concepts [33, 34], and strength training concepts [11, 12, 32, 35, 36]. The planned progression of training for strength, power, and hypertrophy [11, 32, 33, 35, 36] is shown in Table 2.

**Table 2:** Time schedule and progression phases of training for motor learning, strength, hypertrophy, and power for experimental and control groups

Week	Experimental group	Control group	Training frequency
1–5	Motor learning + power	Motor learning	45 (plus 3 personal physiotherapy consultations)
6–9	Strength + hypertrophy + power	Strength + hypertrophy	12 (plus 2 personal physiotherapy consultations)
10–16	Power	Strength + hypertrophy + power	21 (plus 4 personal physiotherapy consultations)

Training procedures for motor learning, strength, hypertrophy, and power training phases follow the training principles of variation/periodization, muscle action and velocity of muscle action, loading, volume, exercise selection, rest period, and frequency [11, 12] for both groups. The training program will last 16 weeks and will contain 9 personal physiotherapy consultations and 78 home training sessions of approximately 15 minutes (weeks 1–5 3x/week, 3x/day; weeks 6–16 3x/week, 1x/day) (Tables 1 and 2). Motor learning and strength and hypertrophy phases are comparable for both groups. However, the main difference between the programs is the applied type of muscle action (control group: isometric, concentric; experimental group: isometric, concentric, eccentric und eccentric-concentric) and speed of movement (control group: voluntary slow to moderate (to explosive) [33, 35]; experimental group: explosive, reactive, reflexive). After completion of the 16-week training program, the participants will participate in no further personal physiotherapy consultations; however, they will continue home training sessions 3x/week until the 6-month follow-up. During the intervention period participants may continue with their individual activities of daily life.

The personal physiotherapy consultations will take place in the physiotherapy center of the Women’s Hospital, Bern University Hospital and University of Bern, Switzerland. The treating physiotherapists are all specialized and experienced in PFM rehabilitation.

An additional document file presents the intervention (therapy plan of intervention group and control group) in detail [see Additional file 1].

#### Adverse events

In the current study there are no anticipated risks or inconveniences, as the applied intervention and examinations are well known and widely applied in pelvic floor standard physiotherapy. The additional exercises of the experimental group are also well known and applied in physiotherapy; however, they have not yet been used with SUI patients.

All the women are asked during every personal physiotherapy and measurement consultation whether they experience any adverse effects.

### Ethical approval

The present randomized controlled trial has been approved by the ethics committee of the Canton of Bern (reference number 249/14 on 12 November 2014). The original title of the study approved by the ethics committee of the Canton of Bern, Switzerland and the Swiss National Science Foundation was: “Stress urinary incontinence physiotherapy: study protocol for a randomized controlled trial with 6-month follow-up.”

A summary flow chart is provided in Fig. 1 (CONSORT study flow diagram).

## Discussion

SUI is increasingly recognized as a health and economic problem which not only troubles the affected women, but also implies a substantial economic burden on health and social services [37]. Physiotherapy, the first-line basic SUI therapy [8, 9], represents only a mere 2 % of total costs [38] and therefore seems to be good value for the money. As activities or efforts typically provoking SUI occur within milliseconds [39, 40], the focus of physiotherapy PFM training protocols on fast and reflexive contractions seems crucial for SUI patients. To the best of the authors’ knowledge, the present study is the first one investigating a PFM protocol focusing on PFM reactivity, that is, investigating involuntary PFM contractions rather than concentrating exclusively on PFM voluntary contractions (standard physiotherapy). Should this newly developed PFM therapy protocol be shown successful, the clinical impact would be high and the application in clinical practice and therefore the benefit for SUI patients immediate. Additionally, the new therapy protocol promises to gain high acceptance by patients as it offers a higher suitability and practicability for daily use, because it has more dynamic whole body movement elements integrated than does standard SUI physiotherapy.

## Trial status

First patient randomized on 11 March 2015

Trial protocol and updates: see clinicaltrials.gov.

## Additional file

Additional file 1:
**Stress Urinary Incontinence Physiotherapy - Therapy Plan.** (PDF 524 kb)

## Abbreviations

ADS: software package “Analoge und digitale Signalverarbeitung”
ANOVA: analysis of variance
BMI: body mass index
CMJ: counter movement jump
DJ: drop jump
EMG: electromyography
ICC: intra-class correlation coefficient
ICIQ-LUTSqol: International Consultation on Incontinence Modular Questionnaire Urinary Incontinence Symptoms Quality of Life
ICIQ-UIsf: International Consultation on Incontinence Modular Questionnaire Urinary Incontinence (short form)
MATLAB: Matrix Laboratory
MD: minimal difference
MVC: maximal voluntary contraction
PFM: pelvic floor muscles
RCT: randomized controlled trial
s: seconds
SEM: standard error of measurement
SJ: squat jump
SUI: stress urinary incontinence
VFC: voluntary fast contraction.
