# Supplementary material for: Involuntary reflexive pelvic floor muscle training in addition to standard training versus standard training alone for women with stress urinary incontinence: study protocol for a randomized controlled trial
Source: Trials. 2015 Nov 17;16:524. doi: 10.1186/s13063-015-1051-0 (PMC4647572; doi:10.1186/s13063-015-1051-0)
Supplement: Additional file 1: — Stress Urinary Incontinence Physiotherapy - Therapy Plan. (PDF 524 kb) [file 13063_2015_1051_MOESM1_ESM.pdf]

Additional file 1. Stress Urinary Incontinence Physiotherapy – Therapy Plan

Stress Urinary Incontinence Physiotherapy – Therapy Plan

(SUIP - Therapy Plan)

Helena Luginbuehl[1,2], Corinne Lehmann [3], Jean-Pierre Baeyens[2], Annette Kuhn[4], Lorenz Radlinger[1]

[1]Bern University of Applied Sciences, Health, Discipline of Physiotherapy, Switzerland

[2]Vrije Universiteit Brussel, Faculty of Physical Education and Physiotherapy, Belgium

[3]Bern University Hospital and University of Bern, Department of Physiotherapy, Switzerland

[4]Women's Hospital, Urogynaecology, Bern University Hospital and University of Bern, Switzerland

Introduction

To date, the focus of research on pelvic floor muscle (PFM) function has been on the concentric and isometric muscle action that leads to the lift and squeeze but so far no light has been shed on the eccentric or eccentric-concentric type of contraction and the related involuntary or reflexive power. “Power” has to be interpreted as mechanical power (*P(t) = F x v* ➔ power equals force times velocity) in the sense of *rate of force development* in the here described context of training.

It can be assumed that the impact loading on the PFM evoked by coughing, running, jumping or any abrupt rise in intra-abdominal pressure provokes involuntary muscle reactivity. Based on the literature review presented in the study protocol the main deficit of an insufficient and incontinent PFM is the lower maximal force and the lower power compared to sufficient PFM. Consequently, the special attribute of the standardized therapy plan for the experimental group introduced here aims at power with voluntary and in contrast to the control group as well as *involuntary reactive strength with reflexive* PFM contractions as a characteristic of a physiological unconscious and not perceived functioning of PFM during daily life activities or activities with short but intensive impacts (jumps, running, coughing etc.).

Although conventional therapy programs finally also focus on the power, however, fast *voluntary* PFM contractions are applied only. That means this program of the control group is carried out without the above mentioned involuntary reactive contractions.

Both programs are based on the latest position stand paper of the American College of Sports Medicine (ACSM) [1, 2], PFM motor learning concepts [3, 4], and strength training concepts [1, 2, 5-7]. However, even in these references the training methods vary within a certain range (e.g. repetitions: 8-12; rest intervals: 1-2 minutes, velocity: slow to moderate, etc.). This lack in standardization of training regimens is well known [8]. Therefore, within in these methodological ranges training programs presented her are precisely standardized for the control and experimental group separately and following the references and training principles and methods.

The planned progression of training for strength, power and hypertrophy is shown in Table 1.

Table 1. Time schedule and progression phases of training for motor learning, strength, hypertrophy and power for experimental and control group

(compiled following [1-3, 5-7])

Week	Experimental group	Control group	Training Frequency
1-5	Motor learning<br>+ power	Motor learning	15 (plus 3 personal physiotherapy consultations)
6-9	Strength + hypertrophy<br>+ power	Strength + hypertrophy	12 (plus 2 personal physiotherapy consultations)
10-16	Power	Strength + hypertrophy<br>+ power	21 (plus 4 personal physiotherapy consultations)

Respected training principles

Training procedures for motor learning, strength, hypertrophy and power training phases will follow the below mentioned training principles for both groups.

Variation / periodization. Changes of a therapy program over time allow for the training stimulus remain to be effective and challenging. The here used classical periodization is characterized by the intention to carry out the fundamental aspects (motor learning, strength, hypertrophy, power) to prepare distinct abilities. [1]

Table 1 shows a 16 weeks lasting training program with a total number of 78 training sessions with additional 9 personal consultations (= physiotherapy sessions) for both groups. Motor learning and strength and hypertrophy phases are comparable for both groups, however the main difference between the programs is the applied type of muscle action (control group (CON): isometric, concentric; experimental group (EXP): isometric, concentric, eccentric und eccentric-concentric) and speed of movement (CON: voluntary slow to moderate (to explosive) [3, 5]; EXP: explosive, reactive, reflexive; for details see tables for weekly training below).

Muscle action and velocity of muscle action. All training adaptations are "specific" to the stimulus applied. The relevant factors are the muscle actions involved (CON: concentric; EXP: concentric, eccentric-concentric), the speed of movement (CON: slow – moderate – quick; EXP: explosive – reactive) and the muscle groups trained (CON: PFM; EXP: PFM). The supra-maximal eccentric actions are known to produce an additional benefit in terms of force generation compared to concentric or isometric contractions.

The recommendations of the ACSM [1] do not bring up the idea of involuntary eccentric-concentric, reactive strength training with maximal explosive contraction velocities as Güllich and Schmidtbleicher [7] propose. However, ACSM recommends jumps with fast repetition velocity for maximal progression in jump performance. This idea will be introduced into the training program of the experimental group aiming on the reactive activation of PFM. The program of the control group uses slow to moderate to quick voluntary contractions instead. Loads during jumps and running are described as a trigger for incontinence.

Loading. Due to the fact that no external weights are possible it is not possible to carry out a 1 repetition maximum (1RM) like recommended. Therefore it is quite difficult to describe the loading of PFM strength training. Voluntary (and involuntary) contractions of the PFM will be performed in relation to maximal voluntary contraction (MVC). The patients will be familiarized with this maximal loading and submaximal loads will be estimated and performed in relation to 100%MVC. [1]

Volume. One to three sets per exercise are recommended to be used by novice individuals. For progression long term studies indicated multiple sets and a systematic variation of volume over time. [1]

Exercise selection. This training program focusses on the voluntary and involuntary PFM contraction. Involuntary PFM contraction will be performed by running or jumping on the spot. [1]

Rest periods. The range of rest periods will be 30 seconds to 120 seconds because the exercises used are with low complexity. [1]

Frequency. Besides 9 personal physiotherapy consultations both groups will train 3 times a week (week 1-5 3x/week, 3x/day; week 6-16 3x/week, 1x/day = 78 home training sessions) for 16 weeks to be comparable in training frequency. [1]

Basic Therapy

The basic therapy will be performed in both groups equally during the 9 intended physiotherapy sessions. Basic therapy represents the usual physiotherapy session contents in the presence of a physiotherapist and patient. Both groups will receive the basic therapy during 9 physiotherapy sessions which homogeneously distributed take place in the weeks 1, 2, 4, 6, 8, 10, 12, 14, and 16.

First, the home exercises and training methods will be instructed, supervised and controlled by the physiotherapist at the 9 physiotherapy sessions only, which is best practice and sufficient [9]. This procedure should also grant a high and sustained adherence over time [10, 11].

Second, each patient will receive the necessary proper basic information and instruction [12] and will be taught to perform a correct voluntary PFM contraction. Basic therapy additionally includes: information regarding anatomical, (patho-)physiological aspects of SUI [13-15], performing an isolated PFM contraction [16], postural control, breathing, and PFM contraction with continuous breathing [17], exercising pre-contraction [18], fluid intake [19], micturition and defecation behavior [20], co-contraction [21] of PFM and M. abdominus transversus [22] during intra-abdominal pressure increase. The 9 personal therapy consultations will not be included into the total amount of 78 therapy units (= 100% of all therapy units).

Third, life style interventions for pelvic floor dysfunction will additionally be given during the therapy and follow-up period following the recommendations of Chiarelli [23].

Therapy plan

According to the above discussed training principles and basic therapy a detailed and standardized training program is carried out in the following for each week of training including a short description of the related physiotherapy session 1-9 (aims, basic therapy) and the separate home exercise program for each group with aims of training, exercises used, relevant muscle action type and contraction velocity, description of loading, number of repetitions, volume, duration of rest, training frequency per day/week, and body position applied.

1st week of exercise program

1st Physiotherapy session

Aims: The patient is able to perform a correct isolated maximal voluntary pelvic floor muscle (PFM) contraction (squeeze around pelvic openings and inward (cranial) lift [24, 25].

Basic therapy: Make an anamnesis and explain the function of the pelvic floor [13-15]. Teach isolated PFM contractions [16] and the home exercise program corresponding to the randomized groups (see below).

Home exercise program			
**Experimental group**		**Control group**	
Aims:	motor learning + power	Aims:	motor learning
Exercises:	isolated PFM contractions	Exercises:	isolated PFM contractions
Muscle action:	concentric-isometric, explosive	Muscle action:	concentric, slow
Loading:	90-100% of MVC	Loading:	60-70% of MVC
Repetitions:	3x 2-3 seconds hold	Repetitions:	8
Volume:	3	Volume:	1
Rest:	60s	Rest:	-
Frequency:	3x/day & 3x/week	Frequency:	3x/day & 3x/week
Position:	supine	Position:	sitting on a armchair

2nd week of exercise program

2nd Physiotherapy session

Aims: Control and improvement of the home exercise program. Patient knows the interaction between diaphragm and PFM. The patient is able to prevent an interruption of breathing while contracting PFM [17].

Basic therapy: Teach the patient to concentrate on breathing. The patient contracts PFM and counts loudly with continuous breathing to five. Teach the home exercise program corresponding to the randomized groups (see below).

Home exercise program			
**Experimental group**		**Control group**	
Aims:	motor learning + power	Aims:	motor learning
Exercises:	isolated PFM contractions	Exercises:	isolated PFM contractions
Muscle action:	concentric-isometric, explosive	Muscle action:	concentric, slow to moderate
Loading:	90-100% of MVC	Loading:	60-70% of MVC
Repetitions:	4x 2-3s hold	Repetitions:	10
Volume:	3	Volume:	1
Rest:	60s	Rest:	-
Frequency:	3x/day & 3x/week	Frequency:	3x/day & 3x/week
Position:	sitting upright (chair without arm)	Position:	supine
Aims:	motor learning + rate of force development	Aims:	motor learning
Exercises:	isolated PFM contractions	Exercises:	isolated PFM contractions
Muscle action:	concentric, explosive	Muscle action:	concentric, slow to moderate
Loading:	90-100% of MVC	Loading:	60-70% of MVC
Repetitions:	3	Repetitions:	8
Volume:	3	Volume:	1
Rest:	60s	Rest:	-
Frequency:	3x/day & 3x/week	Frequency:	3x/day & 3x/week
Position:	sitting upright	Position:	sitting upright (chair without arm)

3rd week of exercise program

Home exercise program

Experimental group		Control group	
Aims:	motor learning + power	Aims:	motor learning
Exercises:	isolated PFM contractions	Exercises:	isolated PFM contractions
Muscle action:	concentric-isometric, explosive	Muscle action:	concentric, slow to moderate
Loading:	90-100% of MVC	Loading:	60-70% of MVC
Repetitions:	5x 4s hold	Repetitions:	10
Volume:	5	Volume:	2
Rest:	60s	Rest:	120s
Frequency:	3x/day & 3x/week	Frequency:	3x/day & 3x/week
Position:	sitting upright (chair without arm)	Position:	supine
Aims:	motor learning + rate of force development	Aims:	motor learning
Exercises:	isolated PFM contractions	Exercises:	isolated PFM contractions
Muscle action:	concentric, explosive	Muscle action:	concentric, slow to moderate
Loading:	90-100% of MVC	Loading:	60-70% of MVC
Repetitions:	5	Repetitions:	8
Volume:	5	Volume:	2
Rest:	60s	Rest:	60s
Frequency:	3x/day & 3x/week	Frequency:	3x/day & 3x/week
Position:	sitting upright	Position:	sitting upright (chair without arm)

4th week of exercise program

3th Physiotherapy Session

Aims: Control and improvement of the home exercise program. The patient is able to perform adequate trunk stabilization in functional positions and movements [17].

Basic therapy: Exercise the correct posture in sitting, standing and lifting. Let feel the difference to inadequate trunk stabilization. Teach the home exercise program corresponding to the randomized groups (see below).

Home exercise program

Experimental group		Control group	
Aims:	motor learning + power	Aims:	motor learning
Exercises:	isolated PFM contractions	Exercises:	isolated PFM contractions
Muscle action:	concentric-isometric, explosive	Muscle action:	concentric, slow to moderate
Loading:	90-100% of MVC	Loading:	60-70% of MVC
Repetitions:	4x 2-3s hold	Repetitions:	10
Volume:	6	Volume:	3
Rest:	60s	Rest:	60s
Frequency:	3x/day & 3x/week	Frequency:	3x/day & 3x/week
Position:	standing upright	Position:	sitting upright
Aims:	motor learning + power	Aims:	motor learning
Exercises:	isolated PFM contractions	Exercises:	isolated PFM contractions
Muscle action:	concentric, explosive	Muscle action:	concentric, slow to moderate
Loading:	90-100% of MVC	Loading:	60-70% of MVC
Repetitions:	5	Repetitions:	8
Volume:	3	Volume:	2
Rest:	60s	Rest:	2 minutes
Frequency:	3x/day & 3x/week	Frequency:	3x/day & 3x/week
Position:	standing upright	Position:	standing upright

5th week of exercise program			
**Home exercise program**			
**Experimental group**		**Control group**	
Aims:	motor learning + power	Aims:	motor learning
Exercises:	isolated PFM contractions	Exercises:	isolated PFM contractions
Muscle action:	concentric-isometric, explosive	Muscle action:	concentric, moderate
Loading:	90-100% of MVC	Loading:	60-70% of MVC
Repetitions:	5x 3-4s hold	Repetitions:	10
Volume:	6	Volume:	3
Rest:	60s	Rest:	60s
Frequency:	3x/day & 3x/week	Frequency:	3x/day & 3x/week
Position:	standing upright	Position:	standing upright
Aims:	motor learning + power	Aims:	motor learning
Exercises:	isolated PFM contractions	Exercises:	isolated PFM contractions
Muscle action:	concentric, explosive	Muscle action:	concentric, moderate
Loading:	90-100% of MVC	Loading:	60-70% of MVC
Repetitions:	3	Repetitions:	8
Volume:	5	Volume:	2
Rest:	60s	Rest:	120s
Frequency:	3x/day & 3x/week	Frequency:	3x/day & 3x/week
Position:	standing upright	Position:	standing in a squat position

6th week of exercise program

4th Physiotherapy session

Aims: Control and improvement of the home exercise program. The patient is able to contract PFM against the resistance of increased intra-abdominal pressure.

Basic therapy: Training of voluntary contraction immediately before coughing or lifting something (The «Knack» = voluntary PFM pre-contraction) [18]. Teach the exercise program corresponding to the randomized groups (see below).

Home exercise program			
**Experimental group**		**Control group**	
Aims:	strength and hypertrophy	Aims:	strength and hypertrophy
Exercises:	isolated PFM contractions	Exercises:	isolated PFM contractions
Muscle action:	concentric, explosive	Muscle action:	concentric-isometric, moderate
Loading:	60-85% of MVC	Loading:	60-70% of MVC
Repetitions:	12	Repetitions:	1x 5s hold
Volume:	3	Volume:	3
Rest:	60s	Rest:	120s
Frequency:	3x/week	Frequency:	3x/week
Position:	standing upright	Position:	sitting on the floor
Aims:	strength and hypertrophy + power	Aims:	strength and hypertrophy
Exercises:	running on the spot with pre-contraction	Exercises:	isolated PFM contractions
Muscle action:	concentric-isometric, reactive	Muscle action:	concentric, quick
Loading:	-	Loading:	60-70% of MVC
Time:	15s	Repetitions:	8
Volume:	3	Volume:	2
Rest:	60s	Rest:	120s
Frequency:	3x/week	Frequency:	3x/week
Position:	standing upright	Position:	sitting on the floor

7th week of exercise program

Home exercise program			
**Experimental group**		**Control group**	
Aims:	strength and hypertrophy	Aims:	strength and hypertrophy
Exercises:	isolated PFM contractions	Exercises:	isolated PFM contractions
Muscle action:	concentric, explosive	Muscle action:	concentric-isometric
Loading:	60-85% of MVC	Loading:	moderate, 60-70% of MVC
Repetitions:	10	Repetitions:	8x 5s
Volume:	3	Volume:	2
Rest:	15s	Rest:	120s
Frequency:	3x/week	Frequency:	3x/week
Position:	standing upright	Position:	lying on the floor in a bridging position
Aims:	strength and hypertrophy + power	Aims:	strength and hypertrophy
Exercises:	running on the spot with pre-contraction	Exercises:	isolated PFM contractions
Muscle action:	concentric-isometric, reactive	Muscle action:	concentric, quick
Loading:	-	Loading:	60-70% of MVC
Time:	15s	Repetitions:	8
Volume:	3	Volume:	3
Rest:	60s	Rest:	120s
Frequency:	3x/week	Frequency:	3x/week
Position:	standing upright	Position:	lying on the floor in a bridging position

Loading = (-): Due to the fact of involuntary contractions the loading cannot be estimated in relation to MVC

8th week of exercise program

5th Physiotherapy session

Aims: Control and improvement of the home exercise program. The patient should be able to create and evaluate a micturition-protocol [19].

Basic therapy: Demonstrate an example of a micturition-protocol. Teach that the micturition and the defecation [20] take place without pressing. Teach the home exercise program corresponding to the randomized groups (see below).

Home exercise program			
**Experimental group**		**Control group**	
Aims:	strength and hypertrophy	Aims:	strength and hypertrophy
Exercises:	isolated PFM contractions	Exercises:	isolated PFM contractions
Muscle action:	concentric, explosive	Muscle action:	concentric-isometric, moderate
Loading:	maximal: 90-100% of MVC	Loading:	60-70% of MVC
Repetitions:	3	Repetitions:	8x 5s
Volume:	5	Volume:	2
Rest:	60s	Rest:	120s
Frequency:	3x/week	Frequency:	3x/week
Position:	standing upright	Position:	lying on forearms and knees
Aims:	strength and hypertrophy + power	Aims:	strength and hypertrophy
Exercises:	running on the spot with pre-contraction	Exercises:	isolated PFM contractions
Muscle action:	concentric-isometric, reactive	Muscle action:	concentric, quick
Loading:	-	Loading:	60-70% of MVC
Time:	15s	Repetitions:	8
Volume:	3	Volume:	3
Rest:	60s	Rest:	120s
Frequency:	3x/week	Frequency:	3x/week
Position:	standing upright	Position:	lying on forearms and knees

Loading = (-): Due to the fact of involuntary contractions the loading cannot be estimated in relation to MVC

9th week of exercise program

Home exercise program			
**Experimental group**		**Control group**	
Aims:	strength and hypertrophy	Aims:	strength and hypertrophy
Exercises:	isolated PFM contractions	Exercises:	isolated PFM contractions
Muscle action:	concentric, explosive	Muscle action:	concentric-isometric, moderate
Loading:	maximal: 90-100% of MVC	Loading:	60-70% of MVC
Repetitions:	4	Repetitions:	10x 5s
Volume:	5	Volume:	2
Rest:	60s	Rest:	120s
Frequency:	3x/week	Frequency:	3x/week
Position:	standing upright	Position:	lying on hands and knees
Aims:	strength and hypertrophy + power	Aims:	strength and hypertrophy
Exercises:	running on the spot with pre-contraction	Exercises:	isolated PFM contractions
Muscle action:	concentric-isometric, reactive	Muscle action:	concentric, quick
Loading:	-	Loading:	60-70% of MVC
Time:	15s	Repetitions:	10
Volume:	3	Volume:	3
Rest:	60s	Rest:	60s
Frequency:	3x/week	Frequency:	3x/week
Position:	standing upright	Position:	lying on hands And knees

Loading = (-): Due to the fact of involuntary contractions the loading cannot be estimated in relation to MVC

10th week of exercise program

6th Physiotherapy session

Aims: Control and improvement of the home exercise program. The patient is able to interpret her micturition-protocol.

Basic therapy: Evaluate and discuss the micturition-protocol. Teach the home exercise program corresponding to the randomized groups (see below).

Home exercise program			
**Experimental group**		**Control group**	
Aims:	power (voluntary)	Aims:	strength and hypertrophy
Exercises:	isolated PFM contractions	Exercises:	isolated PFM contractions
Muscle action:	explosive	Muscle action:	concentric-isometric, moderate
Loading:	-	Loading:	60-70% of MVC
Repetitions:	10	Repetitions:	10x 5s
Volume:	3	Volume:	2
Rest:	60s	Rest:	120s
Frequency:	3x/week	Frequency:	3x/week
Position:	standing upright, squat position	Position:	sitting on the floor with legs crossed
Aims:	power (involuntary)	Aims:	strength and hypertrophy
Exercises:	running on the spot (no pre-contraction)	Exercises:	isolated PFM contractions
Muscle action:	reactive	Muscle action:	concentric, quick
Loading:	-	Loading:	60-70% of MVC
Time:	15s	Repetitions:	12
Volume:	3	Volume:	2
Rest:	60s	Rest:	60s
Frequency:	3x/week	Frequency:	3x/week
Position:	standing upright	Position:	sitting on the floor with legs crossed

Loading = (-): Due to the fact of involuntary contractions the loading cannot be estimated in relation to MVC

11th week of exercise program

Home exercise program			
**Experimental group**		**Control group**	
Aims:	power (voluntary)	Aims:	strength and hypertrophy
Exercises:	isolated PFM contractions	Exercises:	isolated PFM contractions
Muscle action:	explosive	Muscle action:	concentric-isometric, moderate
Loading:	-	Loading:	60-70% of MVC
Repetitions:	10	Repetitions:	10x 5s
Volume:	3	Volume:	2
Rest:	60s	Rest:	120s
Frequency:	3x/week	Frequency:	3x/week
Position:	standing upright, squat position	Position:	standing upright
Aims:	power (involuntary)	Aims:	strength and hypertrophy
Exercises:	squat jumps	Exercises:	isolated PFM contractions
Muscle action:	concentric, explosive	Muscle action:	concentric, quick
Loading:	-	Loading:	60-70% of MVC
Repetitions:	10	Repetitions:	12
Volume:	3	Volume:	3
Rest:	60s	Rest:	60s
Frequency:	3x/week	Frequency:	3x/week
Position:	standing upright	Position:	standing upright

Loading = (-): Due to the fact of involuntary contractions the loading cannot be estimated in relation to MVC

12th week of exercise program

7th Physiotherapy session

Aims: Control and improvement of the home exercise program. The patient is able to contract isolated transversus abdominus muscle [22, 26].

Basic therapy: Training of voluntary contraction of lower abdominal muscles as the patient would like to zip too tight pants. Important: no movements within the spine region and breathing should be performed. Teach the home exercise program corresponding to the randomized groups (see below).

Home exercise program			
**Experimental group**		**Control group**	
Aims:	power (voluntary)	Aims:	strength and hypertrophy
Exercises:	isolated PFM contractions	Exercises:	isolated PFM contractions
Muscle action:	explosive	Muscle action:	concentric-isometric, moderate
Loading:	-	Loading:	60-70% of MVC
Repetitions:	10	Repetitions:	8x 8s
Volume:	4	Volume:	2
Rest:	60s	Rest:	60s
Frequency:	3x/week	Frequency:	3x/week
Position:	standing upright, squat position	Position:	standing upright in a straddle position
Aims:	power (involuntary)	Aims:	strength and hypertrophy
Exercises:	squat jumps	Exercises:	isolated PFM contractions
Muscle action:	concentric, explosive	Muscle action:	concentric, quick
Loading:	-	Loading:	60-70% of MVC
Repetitions:	10	Repetitions:	12
Volume:	4	Volume:	3
Rest:	60s	Rest:	60s
Frequency:	3x/week	Frequency:	3x/week
Position:	standing upright	Position:	standing upright in a straddle position

Loading = (-): Due to the fact of involuntary contractions the loading cannot be estimated in relation to MVC

13th week of exercise program			
**Home exercise program**			
**Experimental group**		**Control group**	
Aims:	power (voluntary)	Aims:	strength and hypertrophy
Exercises:	squat jump	Exercises:	isolated PFM contractions
Muscle action:	concentric, explosive	Muscle action:	concentric-isometric, moderate
Loading:	-	Loading:	60-70% of MVC
Repetitions:	10	Repetitions:	8x 5s
Volume:	3	Volume:	2
Rest:	60s	Rest:	60s
Frequency:	3x/week	Frequency:	3x/week
Position:	standing upright, squat position	Position:	single leg standing (left and right)
Aims:	power (involuntary)	Aims:	strength and hypertrophy
Exercises:	counter movement jump	Exercises:	isolated PFM contractions
Muscle action:	eccentric-concentric, explosive	Muscle action:	concentric, quick
Loading:	-	Loading:	60-70% of MVC
Repetitions:	10	Repetitions:	12
Volume:	3	Volume:	3
Rest:	60s	Rest:	60s
Frequency:	3x/week	Frequency:	3x/week
Position:	standing upright	Position:	single leg standing (left and right)

Loading = (-): Due to the fact of involuntary contractions the loading cannot be estimated in relation to MVC

14th week of exercise program

8th Physiotherapy session

Aims: Control and improvement of the home exercise program. The patient is able to contract PFM and transversus abdominus muscle in a co-contraction simultaneously.

Basic therapy: Training of PFM and transversus abdominus muscle co-contraction combined with coughing or other exhaling maneuvers [21]. Teach the home exercise program corresponding to the randomized groups (see below).

Home exercise program			
**Experimental group**		**Control group**	
Aims:	power (involuntary)	Aims:	strength and hypertrophy
Exercises:	counter movement jumps	Exercises:	isolated PFM contractions
Muscle action:	eccentric-concentric, explosive	Muscle action:	concentric-isometric, moderate
Loading:	-	Loading:	60-70% of MVC
Repetitions:	12	Repetitions:	8x 10s
Volume:	3	Volume:	2
Rest:	60s	Rest:	60s
Frequency:	3x/week	Frequency:	3x/week
Position:	standing upright	Position:	standing upright, squat position
Aims:	power (involuntary)	Aims:	power
Exercises:	drop jump	Exercises:	isolated PFM contractions
Muscle action:	eccentric-concentric, explosive	Muscle action:	concentric-static & concentric quick
Loading:	-	Loading:	60-70% of MVC
Repetitions:	12	Repetitions:	8x (5s + 3x)
Volume:	3	Volume:	2
Rest:	60s	Rest:	120s
Frequency:	3x/week	Frequency:	3x/week
Position:	standing upright	Position:	sitting upright

Loading = (-): Due to the fact of involuntary contractions the loading cannot be estimated in relation to MVC

15th week of exercise program

Home exercise program

Experimental group		Control group	
Aims:	power (involuntary)	Aims:	strength and hypertrophy
Exercises:	counter movement jump	Exercises:	isolated PFM contractions
Muscle action:	eccentric-concentric, explosive	Muscle action:	concentric-isometric, moderate
Loading:	-	Loading:	60-70% of MVC
Repetitions:	12	Repetitions:	8x 10s
Volume:	4	Volume:	2
Rest:	60s	Rest:	60s
Frequency:	3x/week	Frequency:	3x/week
Position:	standing upright	Position:	standing upright, squat position
Aims:	power (involuntary)	Aims:	power
Exercises:	drop jump	Exercises:	isolated PFM contractions
Muscle action:	eccentric-concentric, explosive	Muscle action:	concentric, quick
Loading:	-	Loading:	60-70% of MVC
Repetitions:	12	Repetitions:	8x (10s + 3x)
Volume:	4	Volume:	2
Rest:	60s	Rest:	60s
Frequency:	3x/week	Frequency:	3x/week
Position:	standing upright	Position:	single leg standing (left and right)

Loading = (-): Due to the fact of involuntary contractions the loading cannot be estimated in relation to MVC

16th week of exercise program

9th Physiotherapy session

Aims: Control and improvement of the home exercise program. All open questions of the patient relating to SUI and behavior should be resolved. The patient will be informed about the next steps after this training period.

Basic therapy: Give the patient an opportunity to ask questions. Explain the next steps.

Home exercise program

Experimental group		Control group	
Aims:	power (involuntary)	Aims:	strength and hypertrophy
Exercises:	counter movement jump	Exercises:	isolated PFM contractions
Muscle action:	eccentric-concentric, explosive	Muscle action:	concentric-isometric, moderate
Loading:	-	Loading:	60-70% of MVC
Repetitions:	12	Repetitions:	10x 10s
Volume:	5	Volume:	2
Rest:	60s	Rest:	60s
Frequency:	3x/week	Frequency:	3x/week
Position:	standing upright	Position:	standing upright, squat position
Aims:	power (involuntary)	Aims:	power
Exercises:	drop jump	Exercises:	isolated PFM contractions
Muscle action:	eccentric-concentric, explosive	Muscle action:	concentric, quick
Loading:	-	Loading:	60-70% of MVC
Repetitions:	12	Repetitions:	8x (10s + 3x)
Volume:	5	Volume:	2
Rest:	60s	Rest:	60s
Frequency:	3x/week	Frequency:	3x/week
Position:	standing upright	Position:	single leg standing (left and right)

Loading = (-): Due to the fact of involuntary contractions the loading cannot be estimated in relation to MVC

17th week until 6 months follow-up

After the intervention phase the patients receive an information sheet and instruction about the necessary and following home exercise program between the end of therapy and the 6 months follow-up.
Generally patients should continue performing PFM exercises for years after completing their physiotherapy education sessions [27].

Both groups continue their training as described below

Home exercise program			
**Experimental group**		**Control group**	
Aims:	power (involuntary)	Aims:	strength and hypertrophy
Exercises:	counter movement jump	Exercises:	isolated PFM contractions
Muscle action:	eccentric-concentric, explosive	Muscle action:	concentric-isometric, moderate
Loading:	-	Loading:	60-70% of MVC
Repetitions:	6	Repetitions:	10x 10s
Volume:	4	Volume:	2
Rest:	60s	Rest:	60s
Frequency:	3x/week	Frequency:	3x/week
Position:	standing upright	Position:	standing upright, squat position
Aims:	power (involuntary)	Aims:	power
Exercises:	drop jump	Exercises:	isolated PFM contractions
Muscle action:	eccentric-concentric, explosive	Muscle action:	concentric, quick
Loading:	-	Loading:	60-70% of MVC
Repetitions:	5	Repetitions:	8x (10s + 3x)
Volume:	3	Volume:	2
Rest:	60s	Rest:	60s
Frequency:	3x/week	Frequency:	3x/week
Position:	standing upright	Position:	single leg standing (left and right)

Loading = (-): Due to the fact of involuntary contractions the loading cannot be estimated in relation to MVC

Further information

Take care while you are lifting up something: Don’t forget the pre-contraction of the pelvic floor muscle!
Drink about 1½ liter per day and prefer water. 5-6x micturition per day and 0-1x per night are okay.
Be mindful on the correct posture while sitting, standing and lifting.

References

1. American College of Sports Medicine**: American College of Sports Medicine position stand. Progression models in resistance training for healthy adults.** *Med Sci Sports Exerc* 2009, 41(3):687-708
2. Kraemer WJ, Adams K, Cafarelli E, Dudley GA, Dooly C, Feigenbaum MS, Fleck SJ, Franklin B, Fry AC, Hoffman JR, Newton RU, Potteiger J, Stone MH, Ratamess NA, Triplett-McBride T; American College of Sports Medicine: **American College of Sports Medicine position stand. Progression models in resistance training for healthy adults.** *Med Sci Sports Exerc* 2002, 34(2):364-380
3. Bo K, Morkved S: Motor Learning. In: Bo K, Berghmans B, Morkved S, van Kampen M Eds. *Evidence Based Physical Therapy for the Pelvic Floor – Bridging Science and Clinical Practice.* Edinburgh, Churchill Livingstone 2007, p. 113-118
4. Winstein CJ: **Knowledge of results and motor learning - implications for physical therapy.** *Phys Ther* 1991, 71(2):140-149
5. Bo K, Aschehoug A: Strength Training. In: Bo K, Berghmans B, Morkved S, van Kampen M Eds. *Evidence Based Physical Therapy for the Pelvic Floor – Bridging Science and Clinical Practice.* Edinburgh, Churchill Livingstone 2007, p. 119-132
6. Kraemer WJ, Ratamess NA: **Fundamentals of resistance training: progression and exercise prescription.** *Med Sci Sports Exerc* 2004, 36(4):674-688
7. Güllich A, Schmidtbleicher D: **Dimensionen des Kraftverhaltens.** *Orthopädische Praxis* 1999, 35(11):683-687
8. Dumoulin C, Glazener C, Jenkinson D: **Determining the optimal pelvic floor muscle training regimen for women with stress urinary incontinence.** *Neurourol Urodyn* 2011, 30(5):746-753
9. Felicíssimo MF, Carneiro MM, Saleme CS, Pinto RZ, da Fonseca AM, da Silva-Filho AL: **Intensive supervised versus unsupervised pelvic floor muscle training for the treatment of stress urinary incontinence: a randomized comparative trial.** *Int Urogynecol J* 2010 21(7):835-840
10. Borello-France D, Burgio KL, Goode PS, Ye W, Weidner AC, Lukacz ES, Jelovsek JE, Bradley CS, Schaffer J, Hsu Y, Kenton K, Spino C: **Pelvic Floor Disorders Network. Adherence to behavioral interventions for stress incontinence: rates, barriers, and predictors.** *Phys Ther* 2013, 93(6):757-773
11. Alewijnse D, Mesters I: Strategies to enhance adherence and reduce drop out in conservative treatment. In: Bo K, Berghmans B, Morkved S, van Kampen M Eds. *Evidence Based Physical Therapy for the Pelvic Floor – Bridging Science and Clinical Practice.* Edinburgh, Churchill Livingstone 2007, p. 133-146
12. Bo K: **Pelvic floor muscle training in treatment of female stress urinary incontinence, pelvic organ prolapse and sexual dysfunction.** *World J Urol* 2012, 30(4):437-443
13. Ashton-Miller JA, Howard D, DeLancey JO: The functional anatomy of the female pelvic floor. In: Bo K, Berghmans B, Morkved S, van Kampen M Eds. *Evidence Based Physical Therapy for the Pelvic Floor – Bridging Science and Clinical Practice.* Edinburgh, Churchill Livingstone 2007, p.19-34
14. Vodusek DB: Neuroanatomy and neurophysiology of pelvic floor muscles. In: Bo K, Berghmans B, Morkved S, van Kampen M Eds. *Evidence Based Physical Therapy for the Pelvic Floor – Bridging Science and Clinical Practice.* Edinburgh, Churchill Livingstone 2007, p. 35-43
15. Ashton-Miller JA, Howard D, DeLancey JO: **The functional anatomy of the female pelvic floor and stress continence control system.** *Scand J Urol Nephrol Suppl* 2001, (207):1-7
16. Bo K, Larsen S, Oseid S, Kvarstein B, Hagen R, Jorgensen J: **Knowledge about and ability to correct pelvic floor muscle exercises in women with urinary stress incontinence.** *Neurourol Urodyn* 1988, 7(3):261-262
17. Hodges P, Sapsford R, Pengel L: **Postural and respiratory functions of the pelvic floor muscles.** *Neurourol Urodyn* 2007, 26(3):362-371
18. Miller JM, Perucchini D, Carchidi LT, DeLancey JO, Ashton-Miller J: **Pelvic floor muscle contraction during a cough and decreased vesical neck mobility.** Obstet Gynecol 2001, 97(2):255-260
19. Stay K, Dwyer PL, Rosamilia A: **Women overestimate daytime urinary frequency: the importance of the bladder diary.** *J Urol* 2009, 181(5):2176-2180
20. Lubowski DZ, Swash M, Nicholls RJ, Henry MM: **Increase in pudendal nerve terminal motor latency with defaecation straining.** *Br J Surg* 1988, 75(11):1095-1097
21. Sapsford R, Hodges P, Richardson C, Cooper D, Markwell S, Jull G: **Co-activation of the abdominal and pelvic floor muscles during voluntary exercises.** *Neurourol Urodyn* 2001, 20(1):31-42
22. Bo K, Mørkved S, Frawley H, Sherburn M: **Evidence for benefit of transversus abdominis training alone or in combination with pelvic floor muscle training to treat female urinary incontinence: a systematic review.** *Neurourol Urodyn* 2009, 28(5):368-73
23. Chiarelli P: Lifestyle interventions of pelvic floor dysfunctions. In: Bo K, Berghmans B, Morkved S, van Kampen M Eds. *Evidence Based Physical Therapy for the Pelvic Floor – Bridging Science and Clinical Practice.* Edinburgh, Churchill Livingstone 2007, p. 147-159
24. Henderson JW, Wang S, Egger MJ, Masters M, Nygaard I: **Can women correctly contract their pelvic floor muscles without formal instruction?** *Female Pelvic Med Reconstr Surg* 2013, 19(1):8-12
25. Kegel AH: **Progressive resistance exercise in the functional restoration of the perineal muscles.** *Am J Obstet Gynecol* 1948, 56(2):238-248
26. Bo K, Sherburn M, Allen T: **Transabdominal ultrasound measurement of pelvic floor muscle activity when activated directly or via a transversus abdominis muscle contraction.** *Neurourol Urodyn* 2003, 22(6):582-588
27. Simard C, Tu le M: **Long-term efficacy of pelvic floor muscle rehabilitation for older women with urinary incontinence.** *J Obstet Gynaecol Can* 2010, 32(12):1163-1166
